# The effects of robot-assisted gait training combined with non-invasive brain stimulation on lower limb function in patients with stroke and spinal cord injury: A systematic review and meta-analysis

**DOI:** 10.3389/fnhum.2022.969036

**Published:** 2022-08-16

**Authors:** Wataru Kuwahara, Shun Sasaki, Rieko Yamamoto, Michiyuki Kawakami, Fuminari Kaneko

**Affiliations:** ^1^Department of Rehabilitation Medicine, Keio University School of Medicine, Tokyo, Japan; ^2^Department of Physical Therapy, Graduate School of Health Sciences, Tokyo Metropolitan University, Tokyo, Japan; ^3^Department of Artificial Environment, Safety, Environment and System Engineering, Graduate School of Environment and Information Sciences, Yokohama National University, Kanagawa, Japan

**Keywords:** transcranial direct current stimulation, repetitive transcranial magnetic stimulation, robotics, stroke, spinal cord injury, lower limb

## Abstract

**Objective:**

This study aimed to investigate the effect of robot-assisted gait training (RAGT) therapy combined with non-invasive brain stimulation (NIBS) on lower limb function in patients with stroke and spinal cord injury (SCI).

**Data sources:**

PubMed, Cochrane Central Register of Controlled Trials, Ovid MEDLINE, and Web of Science were searched.

**Study selection:**

Randomized controlled trials (RCTs) published as of 3 March 2021. RCTs evaluating RAGT combined with NIBS, such as transcranial direct current stimulation (tDCS) and repetitive transcranial magnetic stimulation (rTMS), for lower limb function (e.g., Fugl-Meyer assessment for patients with stroke) and activities (i.e., gait velocity) in patients with stroke and SCI were included.

**Data extraction:**

Two reviewers independently screened the records, extracted the data, and assessed the risk of bias.

**Data synthesis:**

A meta-analysis of five studies (104 participants) and risk of bias were conducted. Pooled estimates demonstrated that RAGT combined with NIBS significantly improved lower limb function [standardized mean difference (SMD) = 0.52; 95% confidence interval (CI) = 0.06–0.99] but not lower limb activities (SMD = −0.13; 95% CI = −0.63–0.38). Subgroup analyses also failed to find a greater improvement in lower limb function of RAGT with tDCS compared to sham stimulation. No significant differences between participant characteristics or types of NIBS were observed.

**Conclusion:**

This meta-analysis demonstrated that RAGT therapy in combination with NIBS was effective in patients with stroke and SCI. However, a greater improvement in lower limb function and activities were not observed using RAGT with tDCS compared to sham stimulation.

## Introduction

Many patients with stroke and spinal cord injury (SCI) suffer from motor and gait dysfunction due to lower limb paralysis. It was reported that about 290,000 individuals per year experience a stroke in Japan (Takashima et al., 2017). As common sequelae in stroke patients, mobility deficits associated with lower limb dysfunction seriously affect patients' functional independence and quality of life (van de Port et al., 2006; Tyson et al., 2007). As for SCI, the prevalence ranges from 280 to 1,298 cases per million people (Bickenbach, 2013), with a male-to-female sex ratio of 3–4:1. Similarly, patients with SCI usually have mild to moderate lower limb dysfunction and disability. More than half of such injuries occur before 30 years of age (Sekhon and Fehlings, 2001) and cause subsequent psychological stress to the patient and their caregivers because of disability at such an early age. Patients with lower limb motor paralysis due to stroke and SCI suffer from impaired motor and gait function. Improvement in gait function is strongly desired in patients with lower limb motor paralysis caused by stroke and SCI. However, 6 months after stroke onset, it is estimated that only about 30% of patients can recover to an ambulatory level (Jørgensen et al., 1995). More effective treatment for patients with stroke and SCI is desired.

The application of robotic technology for stroke (Krebs and Hogan, 2006; Oujamaa et al., 2009; Veerbeek et al., 2011; Mehrholz et al., 2020) and SCI (Mehrholz et al., 2012; Cheung et al., 2017) to improve motor function has increased in recent years. Robot-assisted gait training (RAGT) devices utilize electromechanically actuated motors that control movement and exert force on the joints or other parts of the lower limbs and are categorized as end-effector, exoskeleton, mobile, and ankle devices (Oujamaa et al., 2009). However, the efficacy of these devices is still under discussion and yet to be clarified. Some studies have shown a positive effect of RAGT on gait velocity and walking independence in both stroke and SCI (Krebs and Hogan, 2006; Oujamaa et al., 2009; Veerbeek et al., 2011; Mehrholz et al., 2012, 2020; Cheung et al., 2017), while others have found no effect (Mehrholz et al., 2012, 2020). Therefore, careful consideration of means to increase the effectiveness of RAGT is warranted.

Furthermore, as an example of rehabilitation in patients with lower limb motor paralysis, some studies have shown the effectiveness of non-invasive brain stimulation (NIBS), such as transcranial direct current stimulation (tDCS) and repetitive transcranial magnetic stimulation (rTMS). Multiple meta-analyses and systematic reviews have examined the efficacy of tDCS, rTMS, or spinal direct current stimulation on lower limb motor function related to gait and balance function in patients with stroke or SCI (Chieffo et al., 2016; Fleming et al., 2017; Li et al., 2018; Ghayour-Najafabadi et al., 2019; Tung et al., 2019; Abualait and Ibrahim, 2020; Elsner et al., 2020; Gowan and Hordacre, 2020). Although most meta-analyses have demonstrated the effectiveness of NIBS, some have shown limited effectiveness.

Thus, systematic reviews with meta-analyses have verified the effectiveness of RAGT alone or NIBS alone on lower limb motor function in patients with stroke and SCI. The combination of therapeutic exercise and NIBS holds promise for facilitating neuromodulation in subjects with corticospinal-tract lesions (Hiscock et al., 2008; Dimyan and Cohen, 2010; Tanaka et al., 2011; Benito et al., 2012; Sohn et al., 2013; Murray et al., 2015). We hypothesized that RAGT therapy combined with NIBS might be more effective than RAGT combined with sham stimulation in improving lower limb motor function related to gait function in patients with lower limb paralysis. In the present study, we conducted a systematic review with meta-analysis to demonstrate the effects of combined therapy with RAGT and NIBS in patients with stroke and SCI.

## Methods

### Search strategy

The literature review protocol was developed following the Preferred Reporting Items for Systematic Reviews and Meta-Analyses statement (Moher et al., 2009). Articles were assessed and collected from PubMed, Cochrane Central Register of Controlled Trials, Ovid MEDLINE, and Web of Science up to 3 March 2021.

### Study selection

The primary search was conducted using the combined terms: “stroke” OR “spinal cord injury,” “lower limb^*^” OR “lower extremit^*^,” “transcranial magnetic stimulation” OR “transcranial electrical stimulation” OR “transcranial direct current stimulation” AND “robot^*^” OR “orthos^*^” OR “orthotic” OR “automat^*^” OR “computer-aided” OR “computer-assisted” OR “power-assist^*^.”

Additionally, the following parameters were used: clinical trials, randomized controlled trials (RCTs), and scientific articles written in English, with full text available. The publication date was not restricted; additional studies were identified by manual search, and duplicates were removed.

The inclusion criteria were as follows: (1) adult participants (age > 18 years); (2) outcomes, including the effects of NIBS, such as tDCS or rTMS, on lower limb motor function, RCT or randomized controlled crossover trial; (3) recruitment of more than five patients; (4) the same interventions between the experimental and control groups, except for tDCS or rTMS treatment in the experimental group; and (5) studies published in English. The exclusion criteria were as follows: (1) non-peer-reviewed articles and articles only describing protocols; (2) studies that met the eligibility criteria but lacked outcomes owing to a lack of e-mail reply from the author; (3) studies of patients with lower limb dysfunction which was caused by other diseases such as Parkinson's disease or amyotrophic lateral sclerosis.

Concerning the reference selection process and inclusion and exclusion criteria above, the potential articles were screened by two reviewers to remove irrelevant studies. Potentially eligible studies were chosen from the remainder if the full text was available.

From the final selected studies, data on the study design and subjects, intervention, methods, and reporting of information on the effectiveness of RAGT combined with NIBS were extracted from the selected articles to provide information on the results and effects that would be useful in clinical practice (Table 1).

**Table 1:** Characteristics of the included trials.

**Author, year**	**Participants**	**Participants' motor** **functional ability at** **baseline as inclusion** **criteria**	**Training period and** **protocol (duration of** **the intervention,** **session)**	**Robotic setting**	**NIBS setting**	**Outcomes** **used in** **meta-analysis**
Seo et al. (2017)	21 chronic stroke patients (time since onset > 6 months) unilateral hemiplegia with age ≥ 18 IG: *n* = 10 CG: *n* = 11 (Follow up; IG: *n* = 8, CG: *n* = 9)	Gait impairment with a FAC score ≤ 4	10 sessions (20 min/day, every weekday for 2 weeks) of tDCS before RAGT	Walkbot_S Speed: 1.2–18 km/h	tDCS The anodal electrode was placed over the presumed leg area of the lesioned hemisphere, and the cathode was placed on the forehead above the contralateral orbit. IG: 20 min of tDCS at an intensity of 2 mA CG: sham tDCS applied for 20 min	FMA, 10 MWT
Danzl et al. (2013)	Eight chronic stroke patients (time since onset > 12 months) IG: *n* = 4 CG: *n* = 4	N/A	12 sessions (three times/week for 4 weeks) of tDCS before RAGT	Lokomat^®^	tDCS The anode was positioned over the cortical motor area controlling the leg, and a cathode was positioned supraorbitally. IG: 20 min of tDCS at an intensity of 2 mA CG: sham tDCS applied for 20 min	SIS-16, 10 MWT
Geroin et al. (2011)	30 chronic stroke patients (time since onset > 12 months) age < 75 years; European Stroke Scale score ≥ 75 and 85 ≥; MMSE score ≥ 24 IG: *n* = 10 CG (sham): *n* = 10 CG (overground): *n* = 10 (excluded from meta-analysis)	Ability to maintain standing position without aids for at least 5 min; ability to walk independently for at least 15 m with the use of walking aids (cane and orthoses).	10 daily sessions (20 min/day, 5 days/week for 2 weeks) of tDCS during RAGT	GT 1 Speed:1.4–18 km/h	tDCS The anodal electrode was placed over the presumed leg area of the lesioned hemisphere, while the cathode was placed above the contralateral orbit of the eye. IG: 7 min of tDCS at an intensity of 1.5 mA CG: sham tDCS applied for 7 min	10 MWT
Kumru et al. (2016a)	24 SCI patients (time since SCI < 1 year) AIS: C or D IG: *n* = 12 CG: *n* = 12	No limitation of passive range of movement in joints	20 sessions (20 min/day, 5 days/week for 4 weeks) of tDCS during RAGT After the above sessions, only robotic gait training lasted for 4 weeks	Lokomat^®^	tDCS The anode was placed over the leg motor cortex and the cathode over the non-dominant supraorbital area. IG: 20 min of tDCS at an intensity of 2 mA CG: sham tDCS applied for 20 min	LEMS, 10 MWT
Kumru et al. (2016b)	31 SCI patients (time since SCI < 6 months) AIS: C or D IG: *n* = 15 CG: *n* = 16 (Follow up; IG: *n* = 14, CG: *n* = 15)	No limitation of passive range of movement in joints	20 sessions (for < 30 min/day, 5 days/week for 4 weeks) of rTMS before RAGT Continuation of RAGT for 4 weeks more without rTMS	Lokomat^®^	rTMS Intensity: 90% RMT in the muscles with the lowest threshold IG: 1,800 pulses over 20 min (2 s duration bursts of 20 Hz (40 pulses/burst) with intertrain intervals of 28 s) CG: sham rTMS (the double cone coil was held over the vertex as with real real rTMS, but it was disconnected from the main stimulator unit).	LEMS, 10 MWT

IG, intervention group; CG, control group; SCI, spinal cord injury; tDCS, transcranial direct current stimulation; rTMS, repetitive transcranial magnetic stimulation; MMSE, mini mental state examination; RAGT, robot-assisted gait training; RMT, resting motor threshold; FMA, Fugl-Meyer Assessment; 10 MWT, 10-m walk test; LEMS, Total motor score from lower extremities of AIS (American Spinal Injury Association impairment scale) clinical exam; SIS-16, Stroke Impact Scale 16.

### Data extraction and quality assessment

The two researchers extracted data regarding the following parameters of the intervention (tDCS or rTMS) and control groups: number of participants, time since onset, severity, motor function at baseline as inclusion criteria, training period and protocol, robotic setting, and outcome measures.

The quality of the studies based on the inclusion and exclusion criteria was assessed using the Physiotherapy Evidence Database (PEDro) scale. The results of the two researchers were compared, and any discrepancies were evaluated by a third researcher and resolved through discussion.

### Outcomes

Several different outcomes were used in each included study. In this study, we performed comprehensive meta-analyses of similar outcomes owing to low participant numbers in each included study. Therefore, the outcomes were classified based on the International Classification of Functioning, Disability, and Health (ICF) into two components: (1) body functions/structures and (2) activities/participation. If multiple outcome assessments of a study were classified in the same domain, the most comprehensive assessment was adopted. Outcomes after the intervention were used for the meta-analysis. If the data were missing, the authors were contacted.

### Data synthesis and analysis

Results were pooled, and meta-analysis was conducted using Cochrane Collaboration's Review Manager software Version 5.4 (RevMan 5.4, The Nordic Cochrane Center, Copenhagen, Denmark). After pooling the software for calculation, the data are expressed using standard mean difference (SMD) and 95% confidence intervals (CI). Statistical heterogeneity was considered high if the *I*^2^ value was > 50%. *P* < 0.05 in the equivalent *z* test was considered statistically significant. In addition, the following subgroup analyses were conducted: (1) according to participants' characteristics: stroke or SCI; and (2) according to the type of NIBS: tDCS or rTMS.

## Results

### Study selection

**Figure 1:**
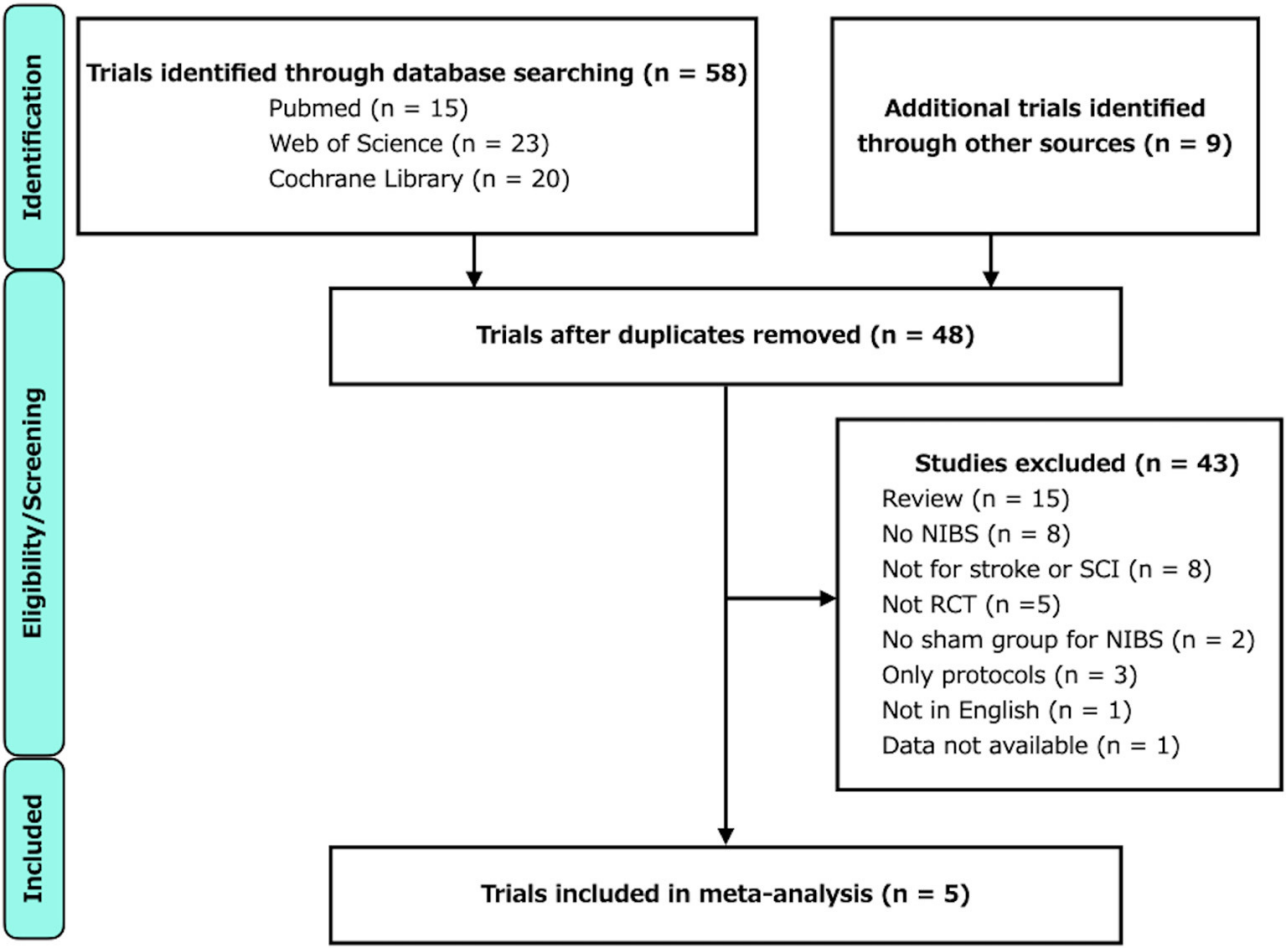
Flowchart for trial selection.

A flowchart of the study selection process is illustrated in Figure 1. The characteristics of the included studies are listed in Table 1. After independent reviews by two researchers, five RCTs comparing the effects of RAGT therapy combined with tDCS or rTMS to sham stimulation on lower limb motor function in patients with stroke and SCI met the eligibility criteria.

### Assessment of risk of bias

For the included RCTs, an assessment of the risk of bias was conducted. The PEDro scale scores for all included studies were between 10 and 11, indicating high quality. The patients were blinded in all studies. Among the studies, there was only one in which therapists were not blinded. Therefore, the potential risk of bias was low. Appropriate randomization was performed in all studies except one, in which participants were assigned in order. Allocation concealment and baseline similarity between groups for the most important prognostic indicators appeared to be present in all studies. The assessment results of the risk of bias using the PEDro score are shown in Supplementary Table 1.

### Outcome selection

The five included RCTs used various outcomes (Table 1). Based on the ICF, these outcomes were classified into the following two subgroups: body functions and activities.

The subgroups of body functions included the following outcomes: (1) Fugl–Meyer assessment of the lower limbs, (2) Stroke Impact Scale 16, and (3) total motor score from the lower extremities of the American Spinal Injury Association Impairment Scale clinical examination. The subgroup of activities included the 10 m walk test.

### Meta-analysis

**Figure 2:**
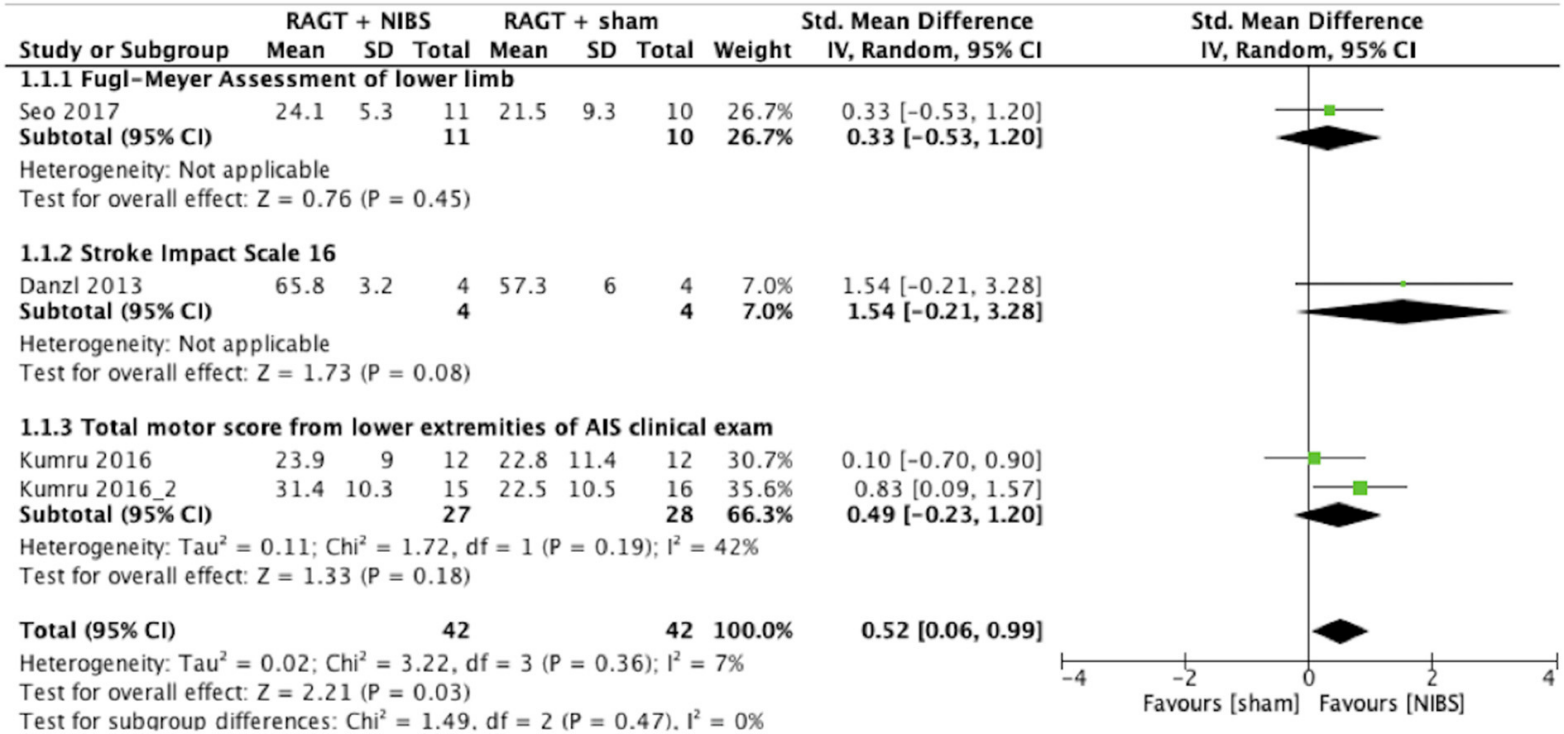
Forest plots: RAGT + NIBS vs. RAGT + sham with outcomes of lower limb body function based on the International Classification of Functioning, Disability, and Health. AIS, American Spinal Injury Association Impairment Scale; NIBS, non-invasive brain stimulation; RAGT, robot assisted gait training.

**Figure 3:**
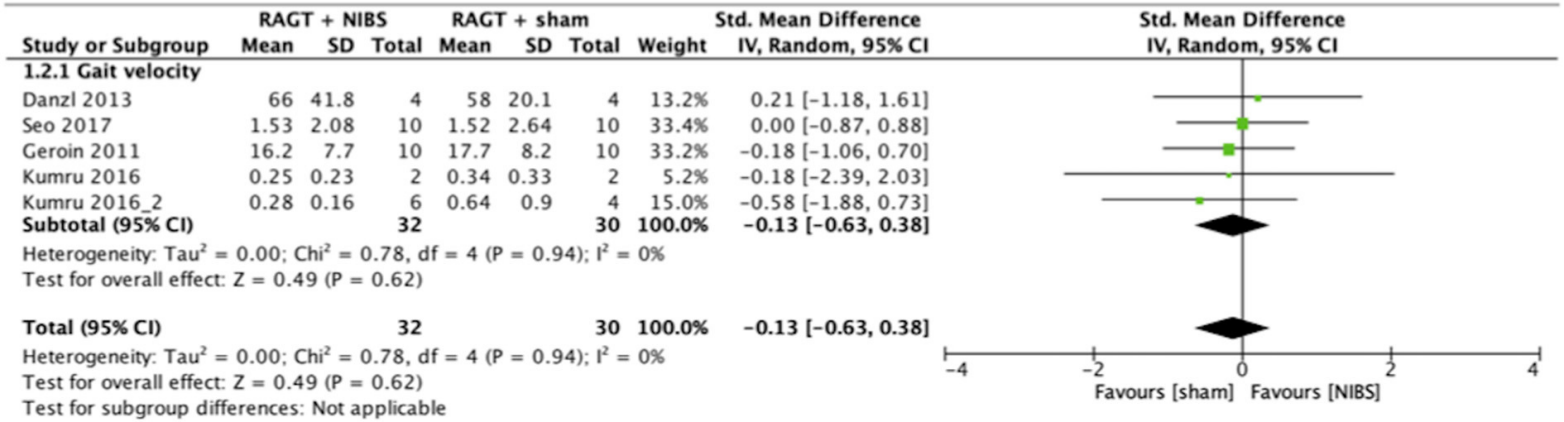
Forest plots: RAGT + NIBS vs. RAGT + sham with outcomes of activities (gait velocity) based on the International Classification of Functioning, Disability, and Health. NIBS, non-invasive brain stimulation; RAGT, robot assisted gait training.

The results of the meta-analysis showed that RAGT with NIBS exhibited a higher effectiveness than sham stimulation regarding lower limb function (SMD = 0.52, 95% CI = 0.06–0.99, *n* = 84, *I*^2^ = 0%; Figure 2) but not regarding lower limb activities (SMD = −0.13, 95% CI = −0.63–0.38, *n* = 62, *I*^2^ = 0%; Figure 3).

The subgroup analyses focused on the participants' characteristics (stroke or SCI) and type of NIBS (tDCS or rTMS) for secondary outcome measures (Table 2). In the function and activities of the lower limbs, while subgroup analyses were performed separately for stroke and SCI, a greater improvement with RAGT with NIBS than with sham stimulation was not observed. A higher level of improvement in lower limb function was observed for RAGT with rTMS than with sham stimulation but not for RAGT with tDCS. In lower limb activities, while subgroup analyses were performed separately for tDCS and rTMS, a greater improvement with RAGT with NIBS than with sham stimulation was not observed. The subgroup analysis showed no significant differences between participants' characteristics or types of NIBS for body function and activities of the lower limbs.

**Table 2:** Subgroup analysis of participants' characteristics (stroke or SCI) and type of NIBS (tDCS or rTMS).

**Subgroup**	**Studies**	**Participants**	**SMD [95% CI]**	***P***	***I^2^***
**1. Body function - participants' characteristics**					
Stroke	2	29	0.69 [−0.39–1.76]	0.23	32%
SCI	2	55	0.49 [−0.23–1.20]	0.19	42%
Subgroup differences				0.76	0%
**2. Body function - type of NIBS**					
tDCS	3	53	0.35 [−0.21–0.90]	0.34	7%
rTMS	1	31	0.83 [0.09–1.57]^a^	0.03^b^	N/A
Subgroup differences				0.30	6.4%
**3. Activities and participation - participants' characteristics**					
Stroke	3	48	−0.10 [−2.09–1.90]	0.87	0%
SCI	2	14	−0.17 [−0.64–0.31]	0.61	0%
Subgroup differences				0.95	0%
**4. Activities and participation - type of NIBS**					
tDCS	4	52	−0.05 [−0.60–0.50]	0.97	0%
rTMS	1	10	−0.58 [−1.88–0.73]	0.39	N/A
Subgroup differences				0.47	0%

CI, confidence interval; SMD, standardized mean difference; SCI, spinal cord injury; NIBS, non-invasive brain stimulation; tDCS, transcranial direct current stimulation; rTMS, repetitive transcranial magnetic stimulation.

a Indicates a significant difference in the comparison between robot-assisted gait training combined with rTMS and sham groups in the equivalent z-test.

b Indicates the P-value of the equivalent z test, which is the result of meta-analysis between robot-assisted gait training combined with rTMS and sham groups.

### Statistical heterogeneity

The statistical heterogeneity of all outcome measures was low to moderate (*I*^2^ < 50%) in this study (Figures 2, 3; Table 2). Therefore, the results of these meta-analyses were considered reliable.

### Adverse effects

Two studies reported adverse effects. One study reported that eight subjects had slightly uncomfortable twitching of the facial muscles or speaking difficulty because of facial-muscle contraction during rTMS, and one subject had a mild headache 1 h after the first rTMS session. The other study reported that one subject in the tDCS group dropped out because of side effects, but no details were provided. Only one study did not report adverse effects.

## Discussion

The present study demonstrated that RAGT therapy combined with NIBS had a significantly better effect compared to RAGT combined with sham stimulation on lower limb body function but not activities (gait velocity) in patients with stroke and SCI. However, in terms of the type of NIBS, there were no significant differences between tDCS and rTMS in body function and lower limb activities, and a greater improvement in lower limb function was not observed using RAGT with tDCS than with sham stimulation. The review showed that three of the five studies applied NIBS immediately before RAGT as pre-conditioning for after-effects (Danzl et al., 2013; Kumru et al., 2016b; Seo et al., 2017). Furthermore, four of the five studies used tDCS as the NIBS intervention (Geroin et al., 2011; Danzl et al., 2013; Kumru et al., 2016a; Seo et al., 2017). That is, the results of this review alone are insufficient to determine the efficacy of rTMS immediately before RAGT for patients with lower limb paralysis in improving lower limb function and activities.

The combination of therapeutic exercise and NIBS such as tDCS (Tanaka et al., 2011; Sohn et al., 2013; Murray et al., 2015) and rTMS (Hiscock et al., 2008; Dimyan and Cohen, 2010; Benito et al., 2012) holds promise for facilitating neuromodulation in subjects with corticospinal tract lesions. It is suggested that these effects are based on the principle of activity-dependent neuroplasticity. Therefore, the mechanism for the positive effect of combining the two different modalities could be a compound effect. The integrity and function of neural networks depend on sustained sensory input and, when interrupted, the brain undergoes multiple processes to correct the disruption (Dayan and Cohen, 2011; Hosp and Luft, 2011). Neuroplasticity leads to changes that facilitate the restoration of sensorimotor integration and output (Jenkins and Merzenich, 1987; Wall et al., 2002). These phenomena underlie mechanisms for long-term potentiation of motor learning and sensorimotor remapping in viable brain regions (Chen et al., 2011; Dalise et al., 2014). That is, NIBS might enhance sensorimotor functions by acting on these neuroplastic mechanisms. Both motor training and NIBS are associated with morphological dendritic plasticity changes within the primary motor cortex (M1) and cognitive brain areas, and these effects might explain the efficacy of combined treatment (Barbati et al., 2020; Cambiaghi et al., 2020, 2021).

However, in this study, there was no greater improvement in lower limb function and activities using RAGT with tDCS than with sham stimulation in patients with stroke and SCI. In NIBS, tDCS was the primary intervention in this study. In contrast, the effect size of the combination of rTMS and RAGT was large in patients with SCI (Kumru et al., 2016b). In a previous meta-analysis examining the effect of NIBS on patients with stroke, it was reported that the effect size of rTMS (Hedges' *g* = 0.46) was larger than that of tDCS (Hedges' *g* = 0.31) (O'Brien et al., 2018). The present study results also showed that the implementation of rTMS rather than of tDCS before or during RAGT might be effective in enhancing the effect of RAGT, at least in patients with SCI (Kumru et al., 2016b). However, since only one study of RAGT combined with rTMS was included in this meta-analysis, more studies related to RAGT combined with rTMS remain needed. The main significance of this study is therefore that it objectively shows that RAGT combined with tDCS, which has been expected to have effectiveness for lower limb function and activities in patients with stroke and SCI, has no effectiveness.

Applications using tDCS should consider the duration per session, electrode size, number, and placement. One study included in this meta-analysis (Geroin et al., 2011) only applied tDCS for 7 min, which might be too short to generate significant effects. Although this study was also included in the Cochrane review (Elsner et al., 2020) which investigated the effect of tDCS on improving activities of daily living and physical functioning in people after stroke, this is the only study that applied tDCS for <10 min. The guidelines state that shorter tDCS duration has shorter after-effects (Woods et al., 2016). Accordingly, this study was not included in the current meta-analysis of lower limb function due to the lack of outcome. However, it was included in the meta-analysis of activities that may be contributing factors to non-significant effects of tDCS. Furthermore, a previous study reported that smaller electrodes, compared to larger electrodes, may produce more focal current density, which could lead to more effective and localized neural modulation (Bastani and Jaberzadeh, 2013). In another study, the authors applied multi-channel electrodes (Otal et al., 2016). A computational modeling study that compared electrode placements targeting M1 (i.e., electrode placements for all studies included in this meta-analysis) and the cerebellum found that cerebellar stimulation produced substantially higher electric field strengths in the target area compared to M1 stimulation, suggesting that the cerebellum may indeed be a suitable target for tDCS (Gowan and Hordacre, 2020). Future tDCS studies should consider these factors.

Furthermore, more recently, studies on the effects of other NIBS, such as transcranial alternating current stimulation (tACS) and transcranial random noise stimulation (tRNS), have been reported (Terney et al., 2008; Moliadze et al., 2010; Saiote et al., 2013; Inukai et al., 2016; Nakazono et al., 2016). In particular, tRNS has been reported to increase the excitability of cortical primary motor areas more stable than tDCS and tACS (Inukai et al., 2016). Thus, various new NIBS algorithms have been developed. Rather than tDCS, different NIBS algorithms might further improve body function and activities, such as gait and velocity of the lower extremities, if performed during or just before RAGT. No study in which rTMS was applied during RAGT was included in this meta-analysis. Further RCTs are needed because rTMS or various new NIBS implementations during RAGT might be an effective intervention to increase lower motor function in patients with stroke and SCI.

Our study had several limitations that must be considered. It was difficult to compare the outcomes of all the included studies. This was because different studies used different clinical measurements to assess factors such as muscle strength and function and activities of daily living, which hindered their comparison. Thus, we comprehensively conducted the meta-analysis based on the ICF. As a result, statistical heterogeneity was low among the studies. The number of the studies included in the present study was only five RCTs. This might be not satisfactory as a meta-analysis. However, as there is no meta-analysis with including over 100 subjects in previous studies for the areas covered in this study (Byeon, 2020; Shen et al., 2020), the suggestion in the present study must be significant for the practitioners. Although the results of the subgroup analysis focused on the type of NIBS showed that three tDCS studies had no effectiveness and only one rTMS study had effectiveness for lower limb function, the results of this study alone may not be interpretable because of insufficient of studies. If more studies related to RAGT therapy combined with tDCS and rTMS are conducted in the future, it will become clear whether the present subgroup analysis results are due to mechanistic differences; such future studies would further enhance the significance of the findings in this study. This study is valuable for demonstrating this need.

## Conclusion

This systematic review and meta-analysis demonstrated the efficacy of RAGT therapy in combination with NIBS for body function but not for activities of the lower limbs in patients with stroke or SCI with motor paralysis. However, in terms of the type of NIBS, there were no significant differences between tDCS and rTMS in body function and lower limb activities, and a greater improvement in lower limb function was not observed using RAGT with tDCS than with sham stimulation. Since only one study of RAGT combined with rTMS was included in this meta-analysis, additional future studies related to RAGT combined with rTMS or different NIBS implementations such as tACS and tRNS are warranted.

## Data availability statement

The original contributions presented in the study are included in the article/Supplementary material, further inquiries can be directed to the corresponding author/s.

## Author contributions

FK and MK were founded. FK managed the project and resources, supervision, conceptualization, partially writing—review, and editing. WK, SS, and RY contributed to data collection, synthesizing the data, and searching the literature. WK and partially all other authors wrote the article. All authors contributed to revising the manuscript and preparing the final version of the manuscript.

## Funding

This study was supported by the Japan Agency for Medical Research and Development (Grant Nos. JP19he2202005 and JP19he2302006).

## Conflict of interest

Authors FK and MK are the founders of INTEP Inc., a commercial company for the development of rehabilitation devices since July 2020. This company had no association with the device or setup used in the current study. The remaining authors declare that the research was conducted in the absence of any commercial or financial relationships that could be construed as a potential conflict of interest.

## Publisher's note

All claims expressed in this article are solely those of the authors and do not necessarily represent those of their affiliated organizations, or those of the publisher, the editors and the reviewers. Any product that may be evaluated in this article, or claim that may be made by its manufacturer, is not guaranteed or endorsed by the publisher.

## Abbreviations

RAGT: robot-assisted gait training
NIBS: non-invasive brain stimulation
RCT: randomized controlled trial
tDCS: transcranial direct current stimulation
rTMS: repetitive transcranial magnetic stimulation
SMD: standardized mean difference
CI: confidence interval
SCI: spinal cord injury
PEDro scale: physiotherapy evidence database scale
ICF: International Classification of Functioning, Disability, and Health.
