# Supplementary material for: The effects of robot-assisted gait training combined with non-invasive brain stimulation on lower limb function in patients with stroke and spinal cord injury: A systematic review and meta-analysis
Source: Front Hum Neurosci. 2022 Aug 16;16:969036. doi: 10.3389/fnhum.2022.969036 (PMC9426300; doi:10.3389/fnhum.2022.969036)
Supplement: Supplementary file 1 [file Table_1.docx]

**Supplemental digital content**

Supplementary Table 1: PEDro scores of included studies

| Author, Year of Publication | PEDro scale items^a^ | | | | | | | | | | | PEDro score (0-10) |
| --- | --- | --- | --- | --- | --- | --- | --- | --- | --- | --- | --- | --- |
|  | 1 | 2 | 3 | 4 | 5 | 6 | 7 | 8 | 9 | 10 | 11 |  |
| Seo HG, et al. (2017) | + | + | + | + | + | + | + | + | + | + | + | 11 |
| Danzl MM, et al. (2013) | + | + | + | + | + | + | + | + | + | + | + | 11 |
| Geroin C, et al. (2011) | + | + | + | + | + | - | + | + | + | + |  | 10 |
| Kumru H, et al. (2016) | + | + | + | + | + | + | + | + | + | + | + | 11 |
| Kumru H, et al. (2016) | + | + | + | + | + | + | + | + | + | + | + | 11 |

Abbreviations: PEDro, Physiotherapy evidence database.

^a^1. Eligibility criteria and source of participants; and 2. random allocation; 3. concealed participants, and 6. blinded therapists; 7. blinded assessors; 8. adequate follow-up; 9. intention-to-treat analysis, and 10. between-group comparisons; 11. point estimates and variability.

High quality, PEDro score 6–10; fair quality, PEDro score 4–5; poor quality, PEDro score ≤ 3.
